# Editorial: The end of qualia. Do we still need phenomenology in the science of consciousness?

**DOI:** 10.3389/fpsyg.2026.2016636

**Published:** 2026-10-28

**Authors:** Michela Balconi, Valentina Cardella, Richard Greene, Riccardo Manzotti, Gianfranco Pecchinenda, Pietro Perconti, Silvano Zipoli Caiani

**Affiliations:** 1 Universita Cattolica del Sacro Cuore, Milan, Italy; 2 Department of Cognitive Sciences, Psychology, Education and Cultural Studies (COSPECS), University of Messina, Messina, Italy; 3 Weber State University, Ogden, UT, United States; 4 Libera Universita di Lingue e Comunicazione, Milan, Italy; 5 Universita degli Studi di Napoli Federico II, Naples, Italy; 6 Universita degli Studi di Firenze, Florence, Italy

**Keywords:** consciousness, illusionism, mind, phenomenology, qualia

Qualia have played a key role in the development of a science of consciousness. They brought into focus the question of whether explaining the availability of information for reasoning, verbal report, and the control of action would suffice to explain conscious experience. A science confined to access consciousness would have to justify that restriction and explain what became of the experiential dimension. The debate about qualia helped establish experience as something that any adequate account of consciousness must address. Alongside this contribution, qualia acquired a further role. They became central to a challenge to physicalism. The qualitative side of consciousness appeared to possess features that even a complete physical description would necessarily leave out. Criticism of physicalism does not itself entail skepticism about a science of consciousness. Nevertheless, the two have frequently been confused. The difficulty of explaining experience has often been interpreted as evidence of a principled limit to scientific explanation. On this reading, qualia identify something that science is constitutionally unable to explain, and the explanatory gap becomes a reason to doubt the prospects of the entire enterprise.

This Research Topic asks whether that second role may have exhausted its theoretical usefulness. A concept can help make a problem visible while eventually constraining the ways in which it can be investigated. What explanatory work do qualia still perform today? Which assumptions associated with them remain productive, and which deserve reconsideration? These questions also reopen the status of phenomenology: how should disciplined inquiry into experience contribute to consciousness science as its conceptual vocabulary changes? The ten contributions approach these questions from different metaphysical and methodological positions. Their disagreements give the Research Topic its scope. Together, they invite an assessment of the continuing value of qualia through the problems they help formulate and the research they make possible.

Several contributions reopen the metaphysical architecture of the problem. Sanfey treats the simultaneity of observer and observed as both the source of the hard problem and a bridge between subjective and physical descriptions, assigning conscious presence a causal role that sequential observation cannot capture. Rail and Selby identify five invariants, that is, situated spatiality, temporal and multimodal integration, intentionality, and pre-reflective selfhood, and seek their formal unity through the “symintentry” hypothesis. Gärtner and Cordovil challenge the demand that consciousness reduce to microphysical properties. According to their account, strict microphysical grounding is problematic even in physics and chemistry, so phenomenal consciousness may not be scientifically exceptional. These proposals disagree sharply, but each rejects the assumption that the only alternatives are reductive microphysicalism and an isolated phenomenal realm.

The pressure on qualia becomes explicit in Loginov's analysis of Chalmers's Moorean argument against illusionism. The claim that experience possesses an irreducible qualitative character, he argues, is not an ordinary common-sense certainty analogous to Moore's claim that external objects exist. It depends on a specialized philosophical vocabulary, while ordinary descriptions remain open to functional interpretation. Manzotti and Perconti's “*Quining Qualia Again: Ten Reasons*” advances the stronger eliminativist case. Its cumulative arguments target epiphenomenality, the regress of an inner observer, empirical undetectability, epistemic redundancy, unreliable introspection, linguistic reification, phenomenal immediacy, and the cultural survival of residual dualism. The paper does not deny that something appears. It denies that appearing is correctly described by positing private inner properties, proposing instead that experience is the world as it exists in relation to a living body. Other papers revise rather than eliminate the concept. Harris recasts qualia as “query acts”: dynamic processes in which predictive systems interrogate sensory input. This framework preserves the privacy and subjectivity of qualia while connecting phenomenological intentionality to predictive error coding. Gomez moves in another direction. He argues that the traditional focus on color overlooks the more fundamental problem of experienced spatiality. The hard problem may already presuppose perceptual categories generated by the consciousness it seeks to explain. Both contributions replace static qualitative items with processes or structural conditions, while resisting straightforward elimination.

The Research Topic's strongest practical answer to its subtitle comes from Troncoso et al. Combining a face-to-face encounter with micro-phenomenological interviews, intersubjective validation, and quantitative analysis, they identify four temporally unfolding structures of embodied response to another's suffering, from relational disengagement to compassionate balanced support. The study shows that first-person evidence need not be treated as an incorrigible inventory of private contents: carefully elicited reports can disclose diachronic, bodily, motivational, and inter-affective organization that conventional questionnaires miss. Phenomenology here functions as a revisable empirical method. Two further contributions broaden the relational and methodological picture. Luo's mirror-induced origination hypothesis models mental self-consciousness as an evolutionary transformation produced through recursive encounters among brain, body, perceptual attributes, and mirror image. Whatever the eventual empirical fate of its eight-stage model, self-consciousness is treated as an achievement rather than a primitive inner given. Through Vygotsky, von Schulz supplies a historical warning against reducing psychology to stimulus and response: cultural-historical and theoretical interpretation are needed to understand human experience without forcing a choice between idealism and materialism. The Research Topic therefore leaves us with a task of conceptual reassessment. The historical success of qualia in exposing the limitations of an exclusively access-based approach deserves recognition. Their continuing scientific value depends on whether they support explanations or generate questions that research can pursue. Treating the difficulty of explaining experience as a permanent verdict on scientific inquiry risks closing those possibilities prematurely.

Finally, one more possibility deserves to be stated: it may be that the problem of qualia was, in the end, a *pseudoproblem* in Thomas Kuhn's technical sense: a puzzle produced by a paradigm rather than found in nature. For Kuhn, normal science doesn't meet its problems. It makes them, because a paradigm decides in advance what counts as a legitimate question, what an answer should look like, and which leftovers must be explained away. On this reading, qualia were never a datum. Nobody has ever run into a quale the way one runs into a rock, a color or a pain. What we run into is the red apple, the sour lemon, the music in the room. The quale enters the picture only afterwards, when a metaphysical assumption has already split the world in two: on one side a physical world stripped of every quality, and on the other a subject that has to put those qualities back, privately and on the inside. The assumption is older than any experiment and survives every one of them. That is exactly the point. The hard problem didn't come from empirical evidence or from phenomenological description. It came from the seventeenth-century separation of the self from the world, of the subject from the object, that Galileo, Descartes and Locke turned into the default settings of modern thought.

If this is right, the history of qualia looks less like the stubborn resistance of a mystery and more like the familiar course of a paradigm in crisis: anomalies pile up, *ad hoc* hypotheses multiply (epiphenomena, emergent properties, proto-experiential dust spread through the cosmos), and the vocabulary grows more refined as it moves further from any possible test. Kuhn reminds us that some questions are never answered. They are dissolved. Nobody measures the Earth's velocity relative to the ether anymore, and nobody weighs phlogiston. Those questions weren't settled; they became unaskable once the framework that supported them gave way. The same thing could happen to qualia. If we stop assuming a subject closed off from the world, with experience locked inside the skull like a private film, the question of how the brain produces qualities may turn out, like the ether, to have no object. What would be left isn't a smaller mystery but a different task: explaining how the world, in the right physical circumstances, becomes the world we experience. That would be a quieter outcome than winning or losing the qualia debate, and a more radical one. This Research Topic may contribute to that shift, after roughly half a century spent trying to naturalize something the world never contained. The problem would not be solved; it would simply be taken off the agenda.

